# Enhancing ESL learner’s literacy by peer-assisted learning strategy of online English news

**DOI:** 10.3389/fpsyg.2023.1172099

**Published:** 2023-10-30

**Authors:** Wen Gong

**Affiliations:** ^1^School of Foreign Studies, Lingnan Normal University, Zhanjiang, China; ^2^Faculty of Humanities and Social Sciences, City University of Macau, Macao, Macao SAR, China

**Keywords:** peer-assisted learning strategy, online English news, collaboration, literacy, knowledge development

## Abstract

As a result of globalization, countries are collaborating more and sharing more information. People’s perspectives now encompass both domestic and international news. In English as a Second Language (ESL) classes, the appropriate use of rich and vibrant online English news resources gives second language learners (L2 learners) direct access to first-hand, authentic English knowledge through independent reading and digestion, as well as peer-assisted classroom activities. Through weekly peer-assisted sharing of online English news, the goal of this study is to improve L2 learners’ literacy, global competence, and appreciation of cultural diversity. This study examines the participants’ perceptions and feedback using both quantitative and qualitative methods to confirm the efficacy of this pedagogy. After the semester-long experiment, significant differences were found in the English proficiency test. Particularly, the experimental class’s reading and writing scores were significantly higher (*p* < 0.05) than the control class’s, indicating that this pedagogy can effectively improve the literacy of ESL learners. The semi-structured interview revealed that participants were pleased with this instruction and perceived improvements in their language skills. ESL students’ core English literacy has significantly improved by expanding their comprehension of news articles and cultivating a sense of social responsibility.

## 1. Introduction

The COVID-19 pandemic, ongoing cultural clashes, aggravated climate change, and escalating food crises are all overlapping and resonating, affecting the international political and economic order in every way. These global issues have highlighted the importance of staying informed and up-to-date with current events. Online English news provides a direct and authentic source of information on these issues. By reading English news, English as a Second Language (ESL) learners can improve their overall English language skills, broaden their horizons, increase their fundamental English knowledge, practice their English listening, reading, and writing skills (Duong and Nguyen, 2021), develop their cross-cultural communication skills (Tao, 2020), and comprehend the ever-changing world. The news can also help students develop a strong sense of social responsibility, correct values, and a deeper understanding of the article’s theme over time (Coelho and Menezes, 2021). Education practices have become deeply ingrained in the social mega-system as a result of the complexity, uncertainty, volatility, and ambiguity of the social transformation period. In this day and age, when human society has entered the “knowledge explosion” and “information explosion,” newspapers are the media that enable us to participate in global issues, think critically, and receive education on a variety of topics. Online English language teaching resources that are based on authentic English may be more effective at enhancing classroom atmosphere (Dheram and Rani, 2007), enhancing teacher-student interaction, broadening students’ horizons, constructing their knowledge systems, and assisting students in acquiring comprehensive cultural awareness in addition to English language knowledge through the original English news. As a result, students’ overall English literacy can be greatly enhanced. In the meantime, it will help cultivate talents with international perspectives and improve students’ overall English application skills.

According to Bacon and Finnemann (1990), ESL students can sharpen their cognitive awareness, broaden their knowledge of various news channels, and maximize their exposure to the most recent news in current affairs through the authenticity of news media. Collaborative learning in the classroom should be encouraged, as research in the field of brain science (Clark and Dumas, 2015) demonstrates that collaborative working triggers the reward system in the brain, thereby enhancing students’ learning and boosting their enthusiasm for learning. The interactive cooperation between teachers and students while completing a predetermined learning task is known as interpersonal connections in the classroom. Factors such as a sense of safety, fairness, warmth, reciprocity, and classroom rules all have an impact on the effectiveness of interactive cooperation (Hammond et al, 2010; Wang, 2014; Scager et al., 2016; Baniasadi et al., 2023). When combined with a peer-assisted learning strategy, online English news can assist ESL learners in improving their overall English language skills, broadening their horizons, increasing their fundamental English knowledge, practicing their English listening, reading, and writing skills, developing their cross-cultural communication skills, and comprehending the ever-changing world. This approach can also help students develop a strong sense of social responsibility, correct values, and a deeper understanding of the article’s theme over time, thereby enhancing their English literacy.

ESL learners may face difficulties with vocabulary acquisition, which can hinder their understanding of the language. They often struggle with reading comprehension due to the abundance of idioms and figurative language in English texts, density of unfamiliar vocabulary, use of homonyms and synonyms, grammar usage, word order, sentence structure, syntax, etc. may experience language challenges that obstruct their personalities, making them feel anxious, isolated, and embarrassed. Besides, ESL learners may also have a hard time following a text or story, picking out important events, and mastering new concepts in their content-area classes. To address these challenges, incorporating Peer-Assisted Learning Strategies (PALS) into ESL instruction is exceptionally beneficial. College students should learn to be socially responsible and care about current events. Many excellent online English newspapers and websites, including *BBC, CNN, The Economist, Newsweek, Time, China Daily, The New Yorker, BusinessWeek,* and others, are emerging worldwide as a result of the development of the Internet and the intensifying cultural exchange between China and western nations. All of these media make it easier for ESL students to understand British and American culture and gain access to authentic English news online. Because it is a realistic, efficient, ubiquitous, vivid, and practical application of textual material derived from real life, English newspaper reading should play an active role in ESL classes. Exposure to authentic language use through media can improve learners’ listening and comprehension skills, as it provides them with real-life examples of language application (Kozhevnikova, 2014; Yin, 2015). Additionally, the use of media in language learning can increase learners’ motivation (Mohammed and Adam, 2016), as it offers a more engaging and interactive learning experience.

Peer assisted learning strategies (PALS) can serve as a tool, in assisting ESL learners in acquiring the skills and knowledge to communicate effectively and navigate diverse cultural settings, which are integral aspects of global competence. PALS have the potential to enhance language proficiency by focusing on grammar and pronunciation all of which play a role in effective global communication. Additionally PALS can foster collaboration and teamwork skills that are crucial for thriving in cultural environments. Consequently PALS contribute significantly to ESL learners comprehension of competence. Enable them to develop the competencies required for success in an increasingly interconnected world. However there remains uncertainty regarding the effectiveness of utilizing the PALS approach, in English news education for improving ESL students English proficiency. Furthermore little is known about how participants evaluate its impact after utilizing it for a semester. Thus conducting research is imperative to substantiate the efficacy of this method.

## 2. Literature review

Topping and Ehly (1998) first proposed Peer-Assisted learning in 1998. Peer-assisted Learning Strategies, or PALS, is a method in which students help others learn more effectively while also learning more effectively themselves. PALS differs from other more general “cooperative learning” methods in that it includes peer tutoring, peer modeling, peer education, peer counseling, peer monitoring, and peer assessment. Fuchs et al. (2001) describe downward and upward extensions of Peer-Assisted Learning Strategies in reading, which was originally developed for Grades 2–6. Later, Fuchs et al. (2002) examine the effects of a dyadic peer-mediated treatment, Peer-Assisted Learning Strategies, on first-grade children’s mathematics development. Peer-assisted learning strategies were used to boost word recognition, fluency, and reading comprehension in young children, according to Fuchs and Fuchs (2005). Sáenz et al. (2005) assess the effects of Peer-Assisted Learning Strategies, a reciprocal classwide peer-tutoring strategy, on the reading performance of native Spanish-speaking students with learning disabilities and their low-, average-, and high-achieving classroom peers. Previous research has demonstrated the usefulness of PALS in promoting students’ reading performance. Therefore, this study aims to investigate how PALS can enhance the overall language development of ESL learners in China.

According to Moglen (2014), students of English as a second language (ESL) have access to a wide range of vocabularies and sentence structures as a result of the news content’s authenticity, immediateness, and usefulness for language learning in context. Additionally, extra-linguistic information in the news can significantly improve student comprehension. The analysis of the translated English headlines revealed that the EFL students’ main difficulties were grammatically followed by discoursal and lexical types (Karazoun, 2016). By employing an action research framework, Park and Jung (2016) evaluates the effectiveness of a video-based curriculum in motivating EFL learners to learn English. Ahmed (2019) examines the pedagogical role of WhatsApp as one of the mobile-assisted language learning applications in developing motivational levels of Yemeni EFL learners to develop reading and writing skills. Chong (2021) deals with the effectiveness of reading aloud using English TV news to increase the number of opportunities and improve self-confidence in producing English utterances for EFL learners. The majority of the limited research on the use of online news media in English as a second language (ESL) classrooms did demonstrate that news media affected a specific area of learners’ language development (such as vocabulary acquisition, speaking, and literacy instruction). The selection of the materials was suggested by some researchers (Bell, 2003); others looked into how students’ exposure to news on television and radio affected specific language skills like speaking proficiency (Bahrani and Tam, 2011) or vocabulary acquisition (Mohammed and Adam, 2016).

None of the studies reviewed above conduct an empirical investigation into the peer-assisted learning strategy (PALS) of online English news in the ESL scenario of China. Therefore, this study aims to demonstrate how a variety of PALS tasks can be incorporated into the ESL curriculum in a Chinese university to maximize students’ content knowledge and overall English language development. As a form of cooperative learning, PALS helps students become more confident, productive, successful, and cooperative (Spörer and Brunstein, 2009; Ahmad et al., 2016; Kelly, 2019; Mendo-Lázaro et al., 2022; Spence, 2022). ESL students benefit greatly from the support of their classmates. English language learners have the opportunity to practice their linguistic skills in a low-stress setting thanks to the assistance of peers, who can both boost students’ self-assurance (Tsuei, 2012; Strickland, 2013) and serve as language models. This pedagogy will help ESL students develop their global awareness and respect of cultural diversity through collaboration. This study adopts the peer-assisted learning strategy (PALS) in English news teaching in ESL settings. It will explicitly address the two research questions:

*RQ1*: How effectively does the PALS of online English news teaching improve the ESL students’ English proficiency?

*RQ2*: How do the participants rate this method of instruction?

## 3. Materials and methods

### 3.1. Research context

The purpose of this research is to investigate the effectiveness of the PALS of online English news teaching in improving the ESL students’ literacy in China. Additionally, the study aims to explore the participants’ perceptions and feedback on this method of instruction.

To this end, a semester-long experiment will be conducted with two groups of ESL students: an experimental group and a control group. The experimental group will receive instruction using the PALS of online English news teaching, while the control group will receive traditional instruction. Both groups of students will take the National College English Test Band 4 (CET-4) at the end of the semester to collect data for further analysis. In addition, a semi-structured interview was conducted with the participants to gather their perceptions and feedback on the instruction. The interview questions will be designed to explore the participants’ experiences with the PALS of online English news teaching and their perceived improvements in their language skills.

The data collected from the College English Test Band 4 (CET-4) and the semi-structured interview will be analyzed using both quantitative and qualitative methods. The quantitative data will be analyzed using SPSS.

### 3.2. Participants

There were two teachers and two classes in this research. Class 1 was taught by the author, who used PALS to teach 41 sophomores majoring in Special Education (9 males and 32 females), while Class 2 was taught by another teacher who did not use the PALS method to teach 39 sophomores majoring in Special Education (8 males and 31 females). The demographic background data is shown in Table 1. The experimental class was divided into roughly ten groups at the beginning of the semester, with three to four students in each group. Each subject of two classes has been studying English for more than a decade. The t-sample test was used to compare the English scores of the two classes on the College Entrance Examination. There was no significant difference (*p* > 0.05), indicating that the two classes’ initial English levels were roughly comparable.

**Table 1:** Demographic background data.

		Class 1 *N* = 41	Class 2 *N* = 39
Gender	Male	9	8
	Female	32	31

### 3.3. Instruments

Both quantitative and qualitative data were gathered in this study. In the first phase of data collection, the English proficiency test (CET-4) scores of the participants were analyzed. At the end of the semester, all classes, both experimental and controlled, took the CET-4 to see if the PALS of online English news pedagogy was effective. After that, the CET-4 results were processed by SPSS. The College English Test (CET) is a national exam for English as a foreign language that is administered by the People’s Republic of China. It examines the English proficiency of Chinese undergraduate and graduate students. This test is very valid and reliable due to its 26-year existence. Writing, listening, reading, and translating are all parts of the CET-4 exam paper. In the second phase of data collection, the researcher obtained data through semi-structured interviews. 15 randomly chosen subjects extended their full cooperation in an online interview.

A semi-structured interview was used to collect qualitative data at the end of the semester to investigate the participants’ perceptions and opinions about this PALS teaching method. Valuable feedback and suggestions were collected while interviewing.

## 4. Research design

### 4.1. Pre-class period

Before attending *College English* class, the subjects (*N* = 41) in the experimental class were required to visit the following English news websites to independently read the most recent news: www.bbc.com, www.voa.org, www.chinadaily.com.cn, www.i21st.cn, www.newsweek.com, etc. They could pick any news according to their interests and preferences. Each student was required to consider selecting one or two keywords in advance and writing a summary of the main idea of the news. After that, students took turns sharing their news in groups within 20 min of the *College English* class.

### 4.2. In-class period

A good way for students to actively discuss the news with teammates is to retell the main ideas. All members of the team should simultaneously complete a form that includes a summary of the main ideas in the news and key buzzwords. The form was then given to the teacher each week for review at the end of class. Not only were students encouraged to comprehend and analyze the media texts, but they were also encouraged to provide their own reflections or perspectives on the news to comment on and evaluate them.

### 4.3. Post-class period

**Figure 1:**
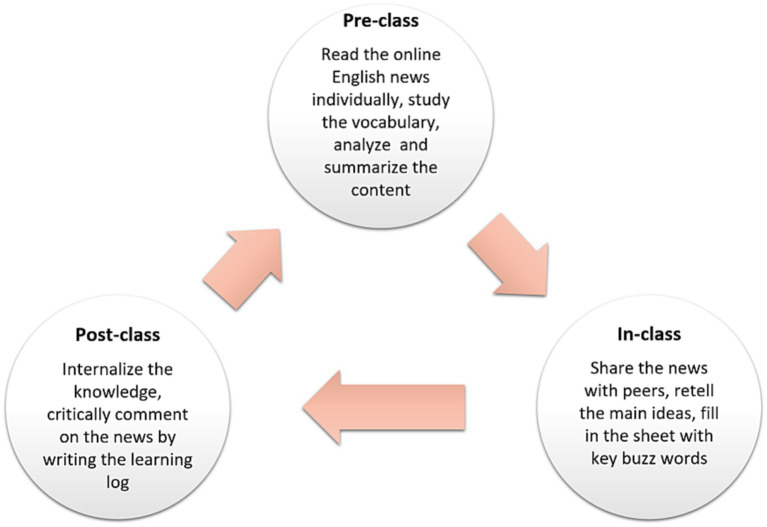
Process of peer-assisted learning strategy of English news.

Students engaged in individual feedback, internalized the information, practiced critical thinking, and summarized the news. After that, the students listed brand-new expressions and vocabulary from these news stories, looked them up, and wrote definitions or explanations for them. They were ultimately required to write a learning log with remarks regarding the news. The cycle of this closed-loop method of instruction lasted 16 weeks (see Figure 1).

**Figure 2:**
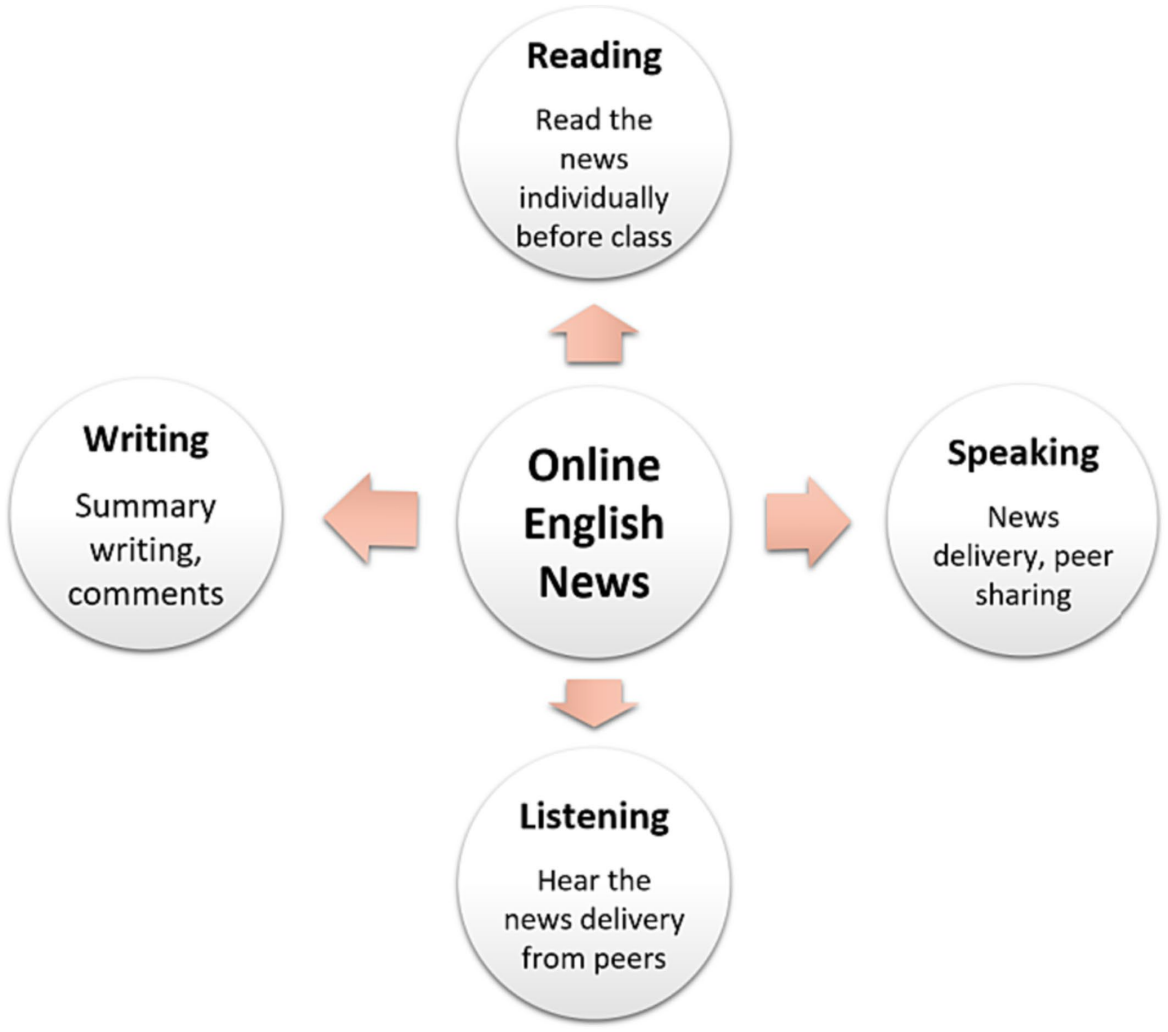
The implementation of integrated English tasks.

The L2 students’ reading, speaking, listening, and writing abilities were fully practiced and developed in various ways. Individual reading allowed students to practice their skills in a one-on-one setting. Peer seminars allowed students to learn from each other and provided a supportive learning environment. In addition to providing students with the opportunity to practice their language skills in a larger group, collective lectures can generate fresh ideas or topics. This combination of individual and group learning strategies allowed the students to practice and develop their language skills comprehensively and effectively. Figure 2 depicts the integration of various language tasks for subjects to acquire content knowledge and improve their language skills simultaneously. Students were given scaffolding to help them not only comprehend new information but also develop complex skills by interacting with a teacher or more experienced classmates. To help their peers with less L2 capability comprehend the content and learn the language, peers with more L2 capability can provide scaffolding.

## 5. Findings

The CET-4 scores of the experimental class (EC) and the controlled class (CC) were collected and compared after 16 weeks of peer-assisted learning of online English news activities. There were significant differences in the average total scores as well as other test scores.

### 5.1. Analysis of the total score

The full score of CET-4 is 710. The average total score of the experimental class (EC) was 464.44, 23.08 points higher than the 441.36 of the controlled class (CC), as shown in Table 2. Students were given complete discretion to gather the most recent news, analyze, and process information in accordance with their interests and preferences. Students’ exploration consciousness and cooperative spirit were enhanced by collaborative English news learning. This encourages students to take the initiative to study and investigate. The development of creativity, which in turn leads to a deeper understanding of society, is facilitated by creating a relaxed and enjoyable atmosphere to pique students’ interest and enthusiasm for English education (Hannah, 2013; Khatimah, 2021; Ramzan et al., 2023).

**Table 2:** Analysis of total score.

Items	*N*	Min	Max	Mean	S.D.	*t*	*p*
Experimental class	41	387.00	552.00	464.44	44.26	67.19	0.00**
Controlled class	39	312.00	515.00	441.36	40.35	48.06	0.00**

*p < 0.05; **p < 0.01.

As shown in Table 2, the minimum total score in EC is 387, which is significantly higher than the score in CC, which is only 312. Similarly, EC’s maximum total score, 552 points, is significantly higher than CC’s (515 points). According to the data, students received a wide variety of high-density linguistic input and the opportunity to demonstrate their language output through self-monitoring, peer sharing, and teacher review. Students made full use of their analytical, synthesizing, critical, and creative skills under the overall supervision, control, and teacher’s guidance, all of which are concrete manifestations of their improved overall English competence.

### 5.2. Analysis of listening score

The listening score of EC was 150.12, which is slightly higher than CC’s score of 146.72, as shown in Table 3. This indicates that subjects significantly improved their listening skills by listening to teammates’ retelling of English news. Team members improved their listening skills as a result of paying close attention to classmates’ weekly oral presentations of English news.

**Table 3:** Analysis of listening score.

Items	*N*	Min	Max	Mean	S.D.	*t*	*p*
Experimental class	41	100.00	210.00	150.12	22.62	42.49	0.00**
Controlled class	39	87.00	200.00	146.72	21.22	36.33	0.00**

*p < 0.05; **p < 0.01.

The comparison of the two classes also reveals a significant difference (*p* = 0.00), as shown in Table 3. The average listening score of EC is 150.12, nearly 4 points higher than that of CC. The subjects’ English listening skills had improved as a result of their regular exposure to online news broadcasts and weekly team members’ news retelling.

### 5.3. Analysis of reading score

Table 4 demonstrates that EC’s average reading score (Mean = 160.83) was nearly ten points higher than the reading score (Mean = 150.44) in CC. Because they were able to better understand news stories through individual comprehension and collaborative learning, this result demonstrates their improved ability to read online news. The students demonstrated their ability to summarize background information, analyze the use of authentic English vocabulary structures, and explore diverse expressions of vocabulary through various sections, including vocabulary acquisition, vocabulary application, background information, and personal reflections. They effectively summarized the main ideas of the texts and approached the topic from different angles and perspectives. Regularly reading the news can help students improve their reading skills by enhancing their comprehension of current social and cultural events, as well as their historical context and cultural impact. Reading news articles can help learners accumulate common and authentic words and phrases, improve their English reading efficiency and level, and gain a broader perspective. Meanwhile, news articles often cover more complex topics, allowing students to progress from pure conversational skills to more academically-focused or business-oriented ones.

**Table 4:** Analysis of reading score.

Items	*N*	Min	Max	Mean	S.D.	*t*	*p*
Experimental class	41	130.00	214.00	160.83	20.32	50.68	0.00**
Controlled class	39	96.00	209.00	150.44	28.46	33.01	0.00**

*p < 0.05; **p < 0.01.

We can easily discover from Table 3 that EC’s minimum score (130 > 96) and maximum score (214 > 209) appeared to be higher than CC’s. Personalized and open-ended online newspaper reading focuses on improving students’ language skills in the acquisition of information, analysis, and problem-solving skills by providing ample opportunities for students to understand, express, and communicate in English. Weekly peer sharing also helps students develop their abilities to learn independently and collaboratively. The latest hot topics in our lives and social development make up the content of English news. On the plus side, it meets university students’ needs for personal growth as well as their emotional and psychological needs. It also expands young students’ access to information, strengthens their sense of responsibility to pay attention to current affairs and political issues, and helps them develop a sense of social responsibility as citizens of the country.

### 5.4. Analysis of writing and translation score

The data in Table 5 show that EC’s writing score was 153.48, which is significantly higher than CC’s score of 144.20. This finding suggests that students were able to use their receptive and productive language skills when completing a variety of integrated tasks, individual internalization, and peer sharing in and out of class by frequently summarizing news stories in oral and written assignments.

**Table 5:** Analysis of writing and translation score.

Items	*N*	Min	Max	Mean	S. D.	*t*	*p*
Experimental class	41	115.00	183.00	153.49	16.96	57.94	0.00**
Controlled class	39	111.00	178.00	144.21	17.97	50.11	0.00**

*p < 0.05; **p < 0.01.

Similar to Table 4, EC’s minimum and maximum scores in Table 5 (115 > 111 and 183 > 178) were higher than CC’s. Promoting L2 writing competence was helped by completing weekly assignments and writing news logs. Students need to search for information in their spare time, comprehend the cultural context of the news, and understand the entire story of the event in order to effectively improve their comprehensive English skills, including reading, writing, and data processing. As a result, the ESL learners’ literacy (or reading and writing skills) significantly improved.

### 5.5. Interview findings

After the semester, fifteen randomly selected students from the experimental class were invited to individual semi-structured interviews. The purpose of the interviews was to ascertain how the participants perceived PAL pedagogy’s contribution to linguistic development. An online conferencing tool was used for each 10-min interview. The 15 interviewees were asked to explain how they felt about the possibility of benefiting from the PAL pedagogy. Regarding the semi-structured interview questions listed in Supplementary material.

#### 5.5.1. Students’ perceived effectiveness of PALS pedagogy

Question 1 inquired about participants’ perceptions of PALS pedagogy and evaluated its efficacy. In the interviews, students made the following comments:

- S1: “I believe that the PALS pedagogy has greatly sparked my interest in English and helped me develop my language skills.”
- S2: “I really like the friendly atmosphere in the peer-assisted learning environment where I can freely share some interesting news with classmates.”
- S3: “I now understand the fundamental elements of news, such as timeliness, conflict, consequence, prominence, proximity, and a lead paragraph’s fundamental elements (5Ws1H).”
- S4: “I know how headlines, captions, sources, lead paragraphs, keywords, and images are arranged in English media texts and journalistic writing.”
- S5: “In various news sections (such as international, politics, sports, health, business, and technology), I’ve picked up new vocabulary and terminologies.”
- S6: “I now know that different news outlets may report the same story from different points of view.”
- S7: “By questioning the news media’s own perspectives on how they support their conclusions, I have learned to be more skeptical of the news.”

The interviewees also stated that they were able to evaluate their work and provide feedback on that of their peers. The majority of students thought that their retelling had significantly increased their knowledge of a variety of news topics and admired the inventiveness of their peers’ work.

The PALS of online English news is beneficial to enhance students’ content knowledge of news as well as their language skills can be perceived as positive and beneficial based on the results of students’ interviews. By reading the news, students understand the common writing feature of news writing (5Ws1H). The 5Ws and 1H of news writing refer to the inverted pyramid style of news writing in which journalists present essential info at the top by answering the who, where, what, when, why, and how questions. Students demonstrated their improved ability to actively engage with the news by analyzing its components, examining how the news story was presented, and providing their summaries and perspectives for the news stories. Students also rekindled their interest in and increased their motivation to contextualize language learning due to the news’s authentic immediacy (Gebhard, 1996). These results suggest that authentic online news, particularly on subjects that students are interested in, can be taught in EFL classes. Students’ content knowledge of various types of news can be effectively enhanced by incorporating authentic online news into appropriate tasks. Students are also exposed to comprehensible language input when lessons focus on comprehension of the news content (5Ws1H) and meaning rather than solely on language forms, such as grammar. This enables students to master the skills and strategies necessary to access and comprehend information.

#### 5.5.2. Students’ perceptions of their language improvements

Concerning the second question, “Which aspect of your English language development do you think you have improved? Why?” The following are a few comments:

- S1: “My reading skills have enhanced significantly as a result of the regular news reading.”
- S2: “The weekly English news retelling has improved my pronunciation and delivery.”
- S3: “My listening greatly improves when I frequently watch online English video news.”
- S4: “My desire to learn English is increased by listening to English news. It increases my interest. When I listen to the same news multiple times, it trains my ears, so I become accustomed to some difficult words. Additionally, extensive listening teaches me the correct pronunciation.”
- S5: “Learning English while listening to the news I enjoy makes learning more fun.”
- S6: “The news in class covered different types of content, such as politics, health, technology, sports, etc. I learned different vocabularies with different kinds of news.”
- S7: “Because I have to write summaries and feedback every week, I am doing better in my English writing.”

Students reported that their vocabulary regarding news had greatly expanded as a result of the variety of news they were interested in. They could actively find the 5Ws1H in the opening paragraph while reading the news. Some students stated that their oral English fluency had significantly improved. The students who were interviewed said that it took a lot of time and effort to multitask, from reading news individually to writing clear news scripts. Many students claim that reading English newspapers weekly allowed them to acquire buzz vocabulary related to current events.

The majority of participants in the interviews reported that they were able to listen attentively to the news content and pay close attention to the fluency and pronunciation of their peers’ news reports. Last but not least, all the interviewees (*N* = 15) stated that they believed their overall English proficiency had increased.

#### 5.5.3. Students’ difficulties and solutions

As for the last question of the semi-structured interview, “What difficulties have you encountered during the semester?” Students were asked about their perceived difficulties and coping strategies during the course. The responses from the interviewees are as follows:

- S1: “At first, I was nervous about doing the weekly oral news report, but after a few weeks of practice, I can now speak more fluently.”
- S2: “I used to be very poor at English listening. After I practice my listening frequently through the peer sharing of English news for a semester, my listening competence greatly improves. Now I can communicate in English fluently and confidently.”
- S3: “The weekly peer sharing of English news, in my opinion, is a lot of fun. We can cover the news that interests us. Additionally, we can assist one another with challenging tasks.”
- S4: “Ah, writing the news log is not an easy task. The news log took a long time to write, edit, and revise.”

Some students reported that if they did not comprehend the material covered in class, they would get access to the website later, watch the news multiple times, or read the headlines of local and international news in Chinese before class. They claimed that their prior knowledge of Chinese helped them better follow up on the English news in class. In addition, it was reported that the learning log, in which students recorded their new vocabulary and composed summaries and reflections, helped them comprehend the news content better. Students noted that the vocabulary work helped them better comprehend how the words were used in the news article and how they could use them in their summary writing.

The majority of students observed that their performance improved with repeated practice and the assistance of their peers in correcting their pronunciation and delivery, although a few students reported that speaking accurately and fluently was frequently difficult for them. 3 participants mentioned that they would feel stressful before the weekly oral presentation of English news. 7 interviewees revealed that they would practice, rehearse their oral script, receive feedback from their peers and adjust as necessary when asked about their coping strategies. They were able to complete the necessary tasks thanks to these strategies, which helped to lessen their apprehension.

## 6. Pedagogical implications for ESL teaching

Being encouraged to read articles about politics and social life, ESL students can pay attention to real-world issues and think deeply. Students who read English magazines and newspapers regularly not only improve their vocabulary and reading fluency, but they also develop cultural confidence, rule awareness, a sense of social responsibility, and a higher cognitive level. News websites, which are products of the information age and serve as a medium for spreading information to a wider demography, have enhanced and made it easier for people to access information. ESL teachers who supervise and guide students in the right way can help L2 students pay attention to current events and develop social responsibility. They interacted with the language with ease and meaning for genuine communication by using their language skills to comprehend the content of the news and respond to it (Oxford, 2002).

In the context of English as a second language (ESL), teachers must make an effort to assist students in better grasping the relevant buzzwords through in-depth exploration and appropriate expansion. The students’ knowledge will be boosted, their awareness of differences and adaptation will be enhanced, they will be assisted in learning to apply relevant translation methods flexibly, their cognitive horizons will be expanded, and their practical application skills will be enhanced as a result of this effort and the training of interpretation quality. When teachers provide them with language materials that include expressions that are frequently used in real life, students are more interested in learning alongside one another and learn very well in English instruction. In contrast, outdated and rigid learning materials have a negative impact on students’ interest in learning. English newspapers and magazines provide learners with adequate language and sociocultural environment training due to the authenticity of the content and the wide range of topics covered.

Establishing a stable collaborative learning relationship often allows students to share information, review prior knowledge, and discuss its connection to new concepts. Students can express an opinion on a topic and talk about the process of completing a task, as well as ask each other questions. This has the benefit of meeting the developmental needs of the student’s social brains. Teachers create scenarios for students to have cooperative learning experiences in the classroom, where cooperative learning enables interaction between multiple students so that students acquire interpersonal and collaborative problem-solving skills and learn to take responsibility for their learning. Students need affirmation and recognition and want others to understand their abilities. A variety of opportunities in the classroom for different categories of students to showcase their talents and charisma can greatly enhance a student’s self-esteem and confidence and motivate them to continue to strive for excellence. The opportunity to showcase oneself is also an opportunity to exercise and improve oneself, participate actively in classroom learning, and provide role models for others to learn from. Each student has unique talents, and when students can share their knowledge and skills with others, not only does the ‘recipient’ benefit from learning something new, but the student acting as a teacher also consolidates knowledge by teaching their knowledge to others.

In the English newspaper reading class, the interactive teacher-student learning mode gives students a rare chance to use their analytical and thinking skills to adaptably solve problems using what they have learned, allowing them to see how fresh, modern, rich, and useful the English news is. Society needs people with solid knowledge, a strong personality, and the ability to think outside the box.

## 7. Conclusion

The study found significant differences (*p* < 0.01) in all scores between the experimental class and the control class through the comparison and analysis of data from the CET-4 results, which sufficiently answers RQ 1. In other words, the study demonstrates that the peer-assisted learning strategy (PALS) of online English news teaching is effective. Self-education is a never-ending process of building up encyclopedic knowledge. Students will be able to acquire as much encyclopedic knowledge as possible for their future workplaces and will be able to keep up with the times by reading the most recent English news if this habit of learning is properly developed during the school years. As for the RQ 2, the semi-structured interview conducted with participants (*N* = 15) revealed that they were pleased with the instruction and perceived improvements in their language skills. Specifically, the core English literacy of ESL students significantly improved by expanding their comprehension of news articles and cultivating a sense of social responsibility. This will have a significant positive impact on the student’s overall quality, professional competence, and career development. Reading the English newspaper frequently and sharing knowledge with peers are two very practical ways to accomplish this. As a result, more schools should be able to use this PALS pedagogy, allowing more ESL practitioners to learn how to use them effectively in and out of the classroom and raising the standard of ESL instruction.

The internet has changed the social structure, encouraging the creation of a three-dimensional space in which human society, physical space, and information space intersect and merge, resulting in a novel social pattern marked by information and resource sharing. A society of talented individuals that encourages the growth of science and technology, which in turn produces qualified teachers for the educational sector, enhancing the educational system and enhancing methods of teaching and learning. Students can develop righteous values by reading English newspapers like *China Daily* and *21st Century*. They can fully mobilize students’ initiative, critical thinking abilities, ability to discover and use the English language through specific learning strategies, and the cultivation of students’ core English literacy by regularly sharing their news reading experience with their peers. They can also help students develop a strong sense of social responsibility, correct values, and a deeper understanding of the article’s theme over time.

In the ESL classroom, strengthening the application of English news can help students improve their overall English language skills as well as broaden their horizons, increase their fundamental English knowledge, practice their English listening, reading, and writing skills, develop their cross-cultural communication skills, and help them comprehend the ever-changing world. The particular application can be used to increase English language training materials by reporting English news, providing listening training, and providing news commentary. A network communication community is built to kindle frequent communications and interactions among learners, giving full play to the overall function of classroom ecology (Gong, 2021). It can also be used to achieve innovation in the English language approach and to improve L2 students’ core English literacy overall, allowing them to use the English language flexibly and laying a solid foundation for their future employment and entrepreneurship.

## Data availability statement

The original contributions presented in the study are included in the article/Supplementary material, further inquiries can be directed to the corresponding author.

## Ethics statement

The study involving human participants were reviewed and approved by Lingnan Normal University Research Ethics Office. Informed consents were obtained from all teacher and student participants of the experimental as well as the control groups in this study.

## Author contributions

The author confirms being the sole contributor of this work and has approved it for publication.
