# Supplementary material for: Enhancing ESL learner’s literacy by peer-assisted learning strategy of online English news
Source: Front Psychol. 2023 Oct 30;14:1172099. doi: 10.3389/fpsyg.2023.1172099 (PMC10642956; doi:10.3389/fpsyg.2023.1172099)
Supplement: Supplementary file 1 [file Presentation_1.pdf]

Appendix I

Semi-structured Interview Questions:	
**1**	What is your perception of the PALS pedagogy? Was is helpful to improve your English proficiency?
**2**	Which aspect of your English language development do you think you have improved? Why?
**3**	What difficulties have you encountered during the semester?

Appendix II

Sample Lesson Plan of PALS Pedagogy (Week 10)

Lesson Title: Unit 4 The Surprising Purpose of Travel
Level: Intermediate
Duration: 90 minutes

Objectives:

- ✧ Develop students' ability to discuss the surprising purpose of traveling in English.
- ✧ Enhance students' reading comprehension skills in English by practicing with travel-related news articles.
- ✧ Foster students' critical thinking skills by enabling them to analyze and discuss travel-related news articles.
- ✧ Improve students' vocabulary and language proficiency by exposing them to authentic materials.

Materials:

- ✧ Textbook (*New Horizon College English* Book 3 from FLTRP).
- ✧ Access to online English news websites, such as *BBC, CNN,Chinadaily, or Newsweek.*
- ✧ Discussion questions related to the news articles.
- ✧ Sheets that include spaces for filling in key buzzwords and summary of the news they read.

Preparatory Task:

- ✧ Ask students to read travel-related news from *BBC, CNN, Chinadaily, Newsweek*, etc.
- ✧ Select some new vocabulary and summarize the main idea of the news they read.

Warm-up (5 minutes):

- ✧ Elicit the topic of travel by showing a short video of travelogue to activate students' prior knowledge.
- ✧ Generate students' interest with photos of beautiful landscapes and souvenirs from around the world.

Presentation (20 minutes):

- ✧ Assign students to read the article "The Surprising Purpose of Travel".
- ✧ Teach the new vocabulary and analyze the structure of the article.
- ✧ Highlight the key points and arguments presented in the article.
- ✧ Ask students to complete a comprehension activity related to the article.

Practice (20 minutes):

- ✧ Encourage students to explore travel-related news beyond the textbook by asking them to share any recent articles or news stories they have read before class.
- ✧ Students take turns sharing the news stories in groups.
- ✧ Provide a brief summary of the story and introduce any new vocabulary they encountered.

- ✧ Encourage other group members to actively listen to the speaker's delivery and ask questions if they have any.
- ✧ Teacher circulates around the classroom, offering assistance and providing feedback to students as needed.

Production (35 minutes):

- ✧ Ask 3 groups to report their discussion and analysis of the news article with the class.
- ✧ Encourage students to ask questions and provide feedback to their classmates.
- ✧ Hand out the sheets to each group for students to fill in the buzzwords and summary, then submit to the teacher.
- ✧ Express appreciation for the students' efforts and encourage them to continue engaging in meaningful discussions.
- ✧ Teacher summarizes the report and highlights the novel ideas that were raised during the discussion.
- ✧ Address any misconceptions or areas that need clarification.

Wrap-up (10 minutes):

- ✧ Review the key vocabulary and language objectives from the text.
- ✧ Highlight several important buzzwords (e.g. outbound, travel blogger, surge, itineraries, long-haul, etc.).
- ✧ Ask students to internalize the knowledge and deepen the understanding of various purposes of traveling.
- ✧ Relate the discussion to the larger context or learning objectives of the lesson.
- ✧ Encourage students to reflect on the different perspectives shared in the discussion.
- ✧ Reinforce the vocabulary by completing the exercises on the textbook.
- ✧ Assign students to write a learning log or comments on the news after class.

This lesson plan is designed for intermediate level ESL learners and incorporates online English news with language teaching. The lesson includes a warm-up, presentation, practice, production, and wrap-up. The materials used include online English news websites, textbook, sheets and discussion questions related to the news articles. The lesson is designed to be interactive and engaging, with opportunities for students to practice reading, speaking, listening, comprehension, discussion and collaboration skills in English.
